# Prognostic importance of the preoperative New‐Naples prognostic score for patients with gastric cancer

**DOI:** 10.1002/cam4.5017

**Published:** 2022-07-14

**Authors:** Hao Wang, Tianyi Fang, Xin Yin, Shenghan Lou, Bangling Han, Jialiang Gao, Yufei Wang, Xibo Wang, Daoxu Zhang, Yimin Wang, Yao Zhang, Yingwei Xue

**Affiliations:** ^1^ Department of Gastroenterological Surgery Harbin Medical University Cancer Hospital, Harbin Medical University Harbin China

**Keywords:** GC, Naples, nomogram, prognosis

## Abstract

**Background:**

The wide applicability of the Naples prognostic score (NPS) is still worthy of further study in gastric cancer (GC). This study aimed to construct a New‐NPS based on the differences in immunity and nutrition in patients with upper and lower gastrointestinal tumors to help obtain an individualized prediction of prognosis.

**Methods:**

This study retrospectively analyzed patients who underwent radical gastrectomy from April 2014 to September 2016. The cutoff values of the preoperative neutrophil‐to‐lymphocyte ratio (NLR), lymphocyte‐to‐monocyte ratio (LMR), serum albumin (Alb), and total cholesterol (TC) were calculated by ROC curve analysis. ROC and t‐ROC were used to evaluate the accuracy of the prognostic markers. The Kaplan–Meier method and log‐rank test were used to analyze the overall survival probability. Univariate and multivariate analyses based on Cox risk regression were used to show the independent predictors. The nomogram was made by R studio. The predictive accuracy of nomogram was assessed using a calibration plot, concordance index (C‐index), and decision curve.

**Results:**

A total of 737 patients were included in training cohort, 411 patients were included in validation cohort. ROC showed that the New‐NPS was more suitable for predicting the prognosis of GC patients. NPS = 2 indicated a poor prognosis. Multivariate analysis showed that CEA (*P* = 0.026), Borrmann type (*P* = 0.001), pTNM (*P* < 0.001), New‐NPS (*P* < 0.001), and nerve infiltration (*P* = 0.035) were independent risk factors for prognosis.

**Conclusion:**

The New‐NPS based on the cutoff values of NLR, LMR, Alb, and TC is not only suitable for predicting prognosis but can also be combined with clinicopathological characteristics to construct a nomogram model for GC patients.

## Graphical abstract

NPS constructed according to the immune and nutritional status of GC patients is more suitable for predicting prognosis

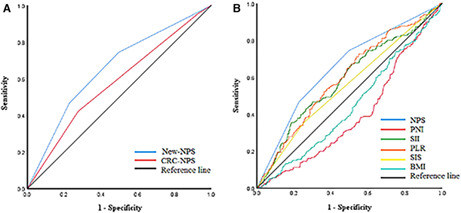

## 1 INTRODUCTION

According to the incomplete statistics of disability‐adjusted life‐years (DALYs) in 2017, gastric cancer (GC) was the third most common malignant tumor, with approximately 860,000 deaths each year.^1^ The incidence rate of malnutrition in patients with malignant tumors is approximately 20%–70%,^2^, ^3^ serious malnutrition may prolong the hospital stay, increase mortality, decrease the treatment effects.^4^, ^5^ Determining the nutritional status of patients before surgery can help predict the risk of death after surgery. However, the definition of malnutrition varies with the type of cancer, and generally, malnutrition is more common in patients with digestive system cancer than in those without digestive system cancer.^6^ In addition, the nutritional status of patients also has obvious heterogeneity due to geographical differences. The Lancet reported that obesity prevalence in Western countries is higher than that in the East, and economic prosperity is an enabler for obesity. However, in some low‐income regions, obesity prevalence is still high.^7^, ^8^ Therefore, it is necessary to individually assess the nutritional status of patients with GC by different regions, which is crucial for predicting patient prognosis.

Galizia et al. first proposed the Naples prognostic score (NPS) for colorectal cancer (CRC), which involves serum albumin (Alb), total cholesterol (TC), neutrophil‐to‐lymphocyte ratio (NLR), and lymphocyte‐to‐monocyte ratio (LMR).^9^ Although the NPS scoring standard of CRC can be used to predict the prognosis GC.^10^ However, GC and CRC are two different solid malignant tumors of the digestive tract, and there may be some differences in the nutritional and immune status of these patients. The proportion of weight loss 2 weeks before surgery in patients with GC is higher than CRC (50% vs. 33%).^11^ This may be due to the occurrence of GC in the upper digestive tract, which may cause dysphagia and malignant obstruction and result in nutrient intake and absorption disorders.^12^ However, the occurrence of CRC in the lower digestive tract has little effect on nutrient intake and absorption.^13^ Although the anatomical distance from the oral cavity to tumor site for CRC is farther than that for GC, the concentration of Fusobacterium in the oral flora is higher in CRC tissue than GC.^14^ The digestive tract flora can regulate tumor microenvironment (TME) immunity and induce proinflammatory gene expression, leading to the occurrence and progression of GC and CRC.^15^ Moreover, the bacterial load in the intestinal tract is also higher than that in the gastral cavity. Additionally, the difference in the concentration of interleukin 7 (IL‐7) secreted by immune cells in the TME between GC and CRC (GC vs CRC: 61.6 pg/g vs. 88.6 pg/g) has been reported to be correlated with the digestive tract flora.^16^ Therefore, the difference in the digestive tract flora may be one of the reasons for the difference between the two in TME immunity.^14^, ^17^ In addition, tumor cells in the TME will cause different degrees of immune activation in the peripheral blood, leading to different degrees of inflammatory response in the body. Previous study found that the cutoff value of systemic immune‐inflammation index (SII) of CRC is different from that of GC (460.66 vs 660).^18^ These differences may cause different NPS standards for CRC and GC. To further detail the individual risk stratification of GC patients and reduce the prognostic bias, we constructed a new NPS (New‐NPS) suitable for GC patients based on the immune and nutritional status of GC patients and predicted the prognostic value of New‐NPS in GC.

This study retrospectively analyzed patients who underwent radical gastrectomy in the Affiliated Tumor Hospital of Harbin Medical University from April 2014 to September 2016 and constructed a new NPS that was more suitable for GC patients.

## 2 MATERIALS AND METHODS

### 2.1 Patients

This study retrospectively analyzed patients who underwent radical gastrectomy at the Affiliated Tumor Hospital of Harbin Medical University from April 2014 to December 2015 as training cohort. Patients who underwent radical gastrectomy at the Affiliated Tumor Hospital of Harbin Medical University from January 2016 to September 2016 were used as the validation cohort. The diagnosis was based on tissue samples obtained from preoperative gastroscopy, which was further confirmed by professional pathologists who analyzed the pathological tissues after surgery. The patients underwent routine preoperative examinations, including abdominal CT, chest CT, echocardiography, double supraclavicular lymph node ultrasound, electrocardiogram, gastroscopy, routine hematology, and tumor markers.

The exclusion criteria of this study were as follows: (1) preoperative chemotherapy, (2) diagnosed with an autoimmune disease, (3) the patient received cholesterol treatment during hospitalization, and (4) hematological malignancies.

The clinicopathological data of the patients were stored in the GC information management system v1.2 of the Affiliated Tumor Hospital of Harbin Medical University (copyright number 2013SR087424). The data included sex, age, body mass index (BMI), carcinoembryonic antigen (CEA), carbohydrate antigen 19–9 (CA19‐9), Borrmann type, tumor diameter, tumor location, pTNM stage, vein invasion, nerve invasion, vascular invasion, lymph node metastasis rate, and postoperative chemotherapy. The above content was in compliance with the eighth edition of AJCC regulations.^19^ The surgical method and lymph node dissection were performed in accordance with the Japanese GC Treatment Guidelines (Fifth Edition).^20^ All patients were followed up after discharge by telephone, email, or check‐up in the Outpatient Complex Building of the the Affiliated Tumor Hospital of Harbin Medical University. The content of the follow‐up included tumor markers, gastroscopy, and abdominal ultrasound. Some patients underwent PET‐CT examinations based on their condition. Patients in stage I were followed up every 12 months, patients in stage II were followed up every 6 months, and patients in stage III were followed up every 3 months.

### 2.2 Hematology sample

The hematology samples of each patient included in this study were collected on an empty stomach in the morning 1 week before surgery. For these samples, 2 ml of blood was collected from the cubital vein and sent to the blood laboratory for testing. The serum was separated for analysis, and the platelet count, lymphocyte count, neutrophil count, monocyte count, serum albumin (Alb), and total cholesterol (TC) were determined. For the inflammation and nutritional indexes, the neutrophil‐to‐lymphocyte ratio (NLR) was calculated as NLR = N/L, the platelet‐to‐lymphocyte ratio (PLR) was calculated as PLR = P/L, the lymphocyte‐to‐monocyte ratio (LMR) was calculated as LMR = L/M, the systemic immune‐inflammation index (SII) was calculated as SII=N × P/L, and the prognostic nutritional index (PNI) was calculated as PNI = 10 × serum albumin (g/dl) + 5 × total lymphocytes (/10^9^) (L = lymphocyte count, N = neutrophil count, M = monocyte count, P = platelet).

### 2.3 Statistical analysis

Overall survival (OS) was defined as the follow‐up time from the time of operation to the time of death or the last survival. If the patient was alive at the last follow‐up, they were included in this study. OS is expressed as the mean ± standard deviation and the 5‐year survival rate. The receiver operating characteristic curve (ROC) was used to calculate and compare the prognostic accuracy of New‐NPS and CRC‐NPS. The diagnostic significance of the inflammation and nutritional indexes for GC and the area under the curve (AUC) were calculated. The optimum cutoff value was calculated by “Youden index”, that is, sensitivity‐(1‐specificity), where the maximum value is the optimum cutoff value. The R package “timeROC” was used for t‐ROC analysis. The chi‐square test was used to analyze the relationship between NPS and patient characteristics. The Kaplan–Meier method and long‐rank test were used to analyze the survival curve. A Cox risk proportional regression model was used to analyze and calculate hazard ratios (HRs) and 95% confidence intervals (CIs) and determine independent risk factors related to patient prognosis. R software was used to construct a nomogram model of risk assessment through the “SvyNom” and “rms” software packages. Calibration plots showed the relationship between predicted probabilities and the actual outcome by using the Hosmer goodness‐of‐fit test. The concordance index is used to measure the accuracy of the nomogram, with bootstrapping to correct for optimistic bias. Decision curves were used to validate the performance of the nomogram for predicting prognosis. All analyses were performed using SPSS for Windows version 25.0 and R software version 4.1.2 for statistical analysis, and *P* < 0.05 was considered statistically significant.

## 3 RESULTS

### 3.1 Patient characteristics

**FIGURE 1:**
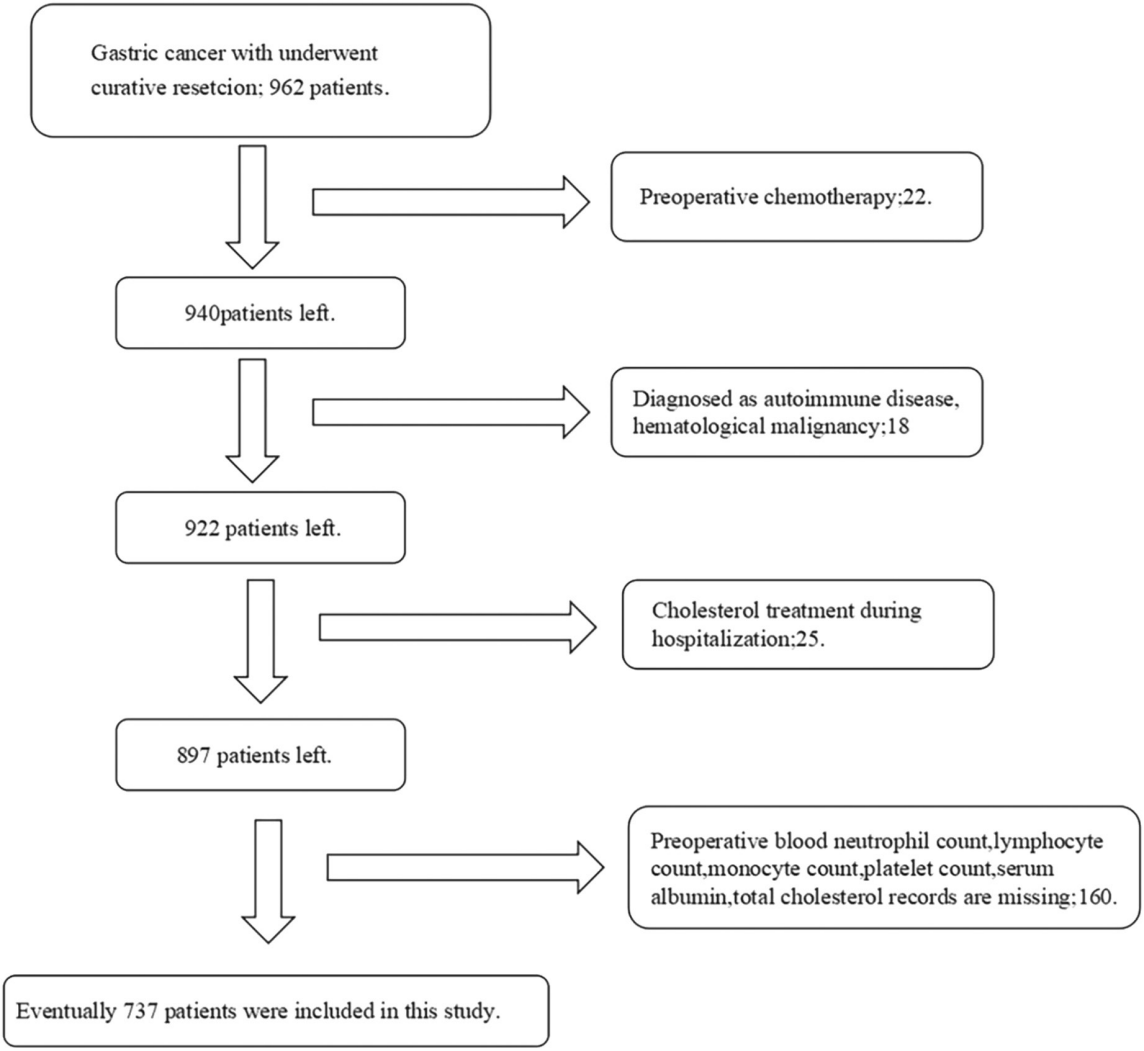
Study protocol design according to the criteria.

After excluding patients according to the exclusion criteria (Figure 1), a total of 737 patients were included in the training cohort (Table 1). The patients included 519 (70.4%) males and 218 (29.6%) females. The mean age of the patients was 57.9 years (range 23–82). According to the pTNM stage, there were 267 (36.2%), 250 (33.9%), and 220 (29.9%) patients in stage I, stage II, and stage III.

**TABLE 1:** Basic characteristics of patients

Characteristics	Training cohort (*n* = 737)	Validation cohort (*n* = 411)	*P* value
Sex			0.970
Male	519 (70.4)	289 (70.3)	
Female	218 (29.6)	122 (29.7)	
Age (years)			0.286
≤60	407 (55.2)	213 (51.8)	
>60	330 (44.8)	198 (48.2)	
BMI (kg/m^2^), median (IQR)	22.8 (20.7–25.2)	23.2 (20.6–25.3)	
CEA, ng/ml			0.119
≤5	635 (86.2)	340 (82.7)	
>5	102 (13.8)	71 (17.3)	
CA19‐9, U/ml			0.298
≤37	667 (90.5)	364 (88.6)	
>37	70 (9.5)	47 (11.4)	
Borrmann type			**<0.001**
0–II	394 (53.5)	155 (37.7)	
III	260 (35.3)	238 (57.9)	
IV	83 (11.3)	18 (4.4)	
Tumor diameter (mm)			0.149
≤50 mm	499 (67.7)	261 (63.5)	
>50 mm	238 (32.3)	150 (36.5)	
Tumor location			0.795
Upper third	101 (13.7)	48 (11.7)	
Middle third	110 (14.9)	61 (14.8)	
Lower third	508 (68.9)	291 (70.8)	
Total stomach	18 (2.4)	11 (2.7)	
Pathological stage			**0.002**
I	267 (36.2)	133 (32.4)	
II	250 (33.9)	114 (27.7)	
III	220 (29.9)	164 (39.9)	
Venous invasion			**0.020**
No	531 (72.0)	269 (65.5)	
Yes	206 (28.0)	142 (34.5)	
Neural infiltration			0.203
No	366 (49.7)	188 (45.7)	
Yes	371 (50.3)	223 (54.3)	
Lymph node ratio (%)			0.052
0	365 (49.5)	172 (41.8)	
0–0.3	256 (34.7)	158 (38.4)	
0.3–0.6	74 (10.0)	57 (13.9)	
>0.6	42 (5.7)	24 (5.8)	
Vascular invasion			0.493
No	482 (65.4)	277 (67.4)	
Yes	255 (34.6)	134 (32.6)	
Postoperative chemotherapy			0.450
No	417 (56.6)	242 (58.9)	
Yes	320 (43.4)	169 (41.1)	

*Note*. Borrmann type and pTNM stage were determined according to the eighth edition of the AJCC Cancer Staging Manual. Tumor location and tumor diameter were determined according to the postoperative pathology report. Statistically significant *P* values are in bold (*P* < 0.05).

Abbreviations: BMI, body mass index; CEA, carcinoembryonciantigen; CA19‐9, carbohydrateantigen19‐9.

A total of 411 patients were included in the validation cohort, including 289 (70.3%) males and 122 (29.7%) females. The mean age of the patients was 59.6 years (range 30–84). According to pTNM stage, there were 133 (32.4%), 114 (27.7%), and 164 (39.9%) patients with stage I, stage II, and stage III (Table 1).

### 3.2 Comparison of the prognostic accuracy of inflammatory and nutritional indexes, New‐NPS, and CRC‐NPS

The NPS, SIS, SII, PLR, NLR, LMR, and PNI were calculated based on the examination results of the patient blood samples obtained 1 week before operation. The systemic inflammation score (SIS) was defined as follows: patients with serum albumin concentration < 40 g/L and LMR <4.44 were given a score of 2; patients with Alb≥40 g/L or LMR≥4.44 were given a score of 1; and patients with Alb≥40 g/L and LMR≥4.44 were given a score of 0.

The optimum cutoff values of the NLR, LMR, Alb, TC, PLR, SII, and PNI were 2.37, 3.10, 42.50, 156.81, 109.74, 720.28, and 50.93. To compare the prognostic accuracy of CRC‐NPS and New‐NPS, the total NPS score of GC patients was calculated according to the scoring standard of Galizia et al. for CRC. Then, the New‐NPS was constructed according to the optimum cutoff values of NLR, LMR, Alb, and TC. For Alb, TC, and LMR, a cutoff value higher than the optimum cutoff value was assigned a score of 0, and a cutoff value lower than the optimum cutoff value was assigned a value of 1 point. For NLR, a score higher than the optimum cutoff value was assigned a score of 1, and a score lower than the optimum cutoff value was assigned a score of 0. Finally, the New‐NPS total score was calculated, and the total score was divided into NPS = 0, NPS = 1, and NPS = 2. The patients were grouped according to the NPS score. The number of patients with NPS = 0, NPS = 1, and NPS = 2 was 326 (44.2%), 200 (27.1%), and 211 (28.6%). The calculation method is listed in Table 2.

**TABLE 2:** Calculation methods of CRC‐Naples and New‐Naples

	CRC‐NPS		New‐NPS	
Factor		Score		Score
Serum albumin, g/dl	≥4	0	≥4.25	0
	<4	1	<4.25	1
Total cholesterol, mg/dl	>180	0	≥156.81	0
	≤180	1	<156.81	1
Neutrophil: lymphocyte ratio	≤2.96	0	≤2.37	0
	>2.96	1	>2.37	1
Lymphocyte: monocyte ratio	>4.44	0	≥3.10	0
	≤4.44	1	<3.10	1
Group	Total score			
NPS 0		0		0 or 1
NPS 1		1 or 2		2
NPS 2		3 or 4		3 or 4

**FIGURE 2:**
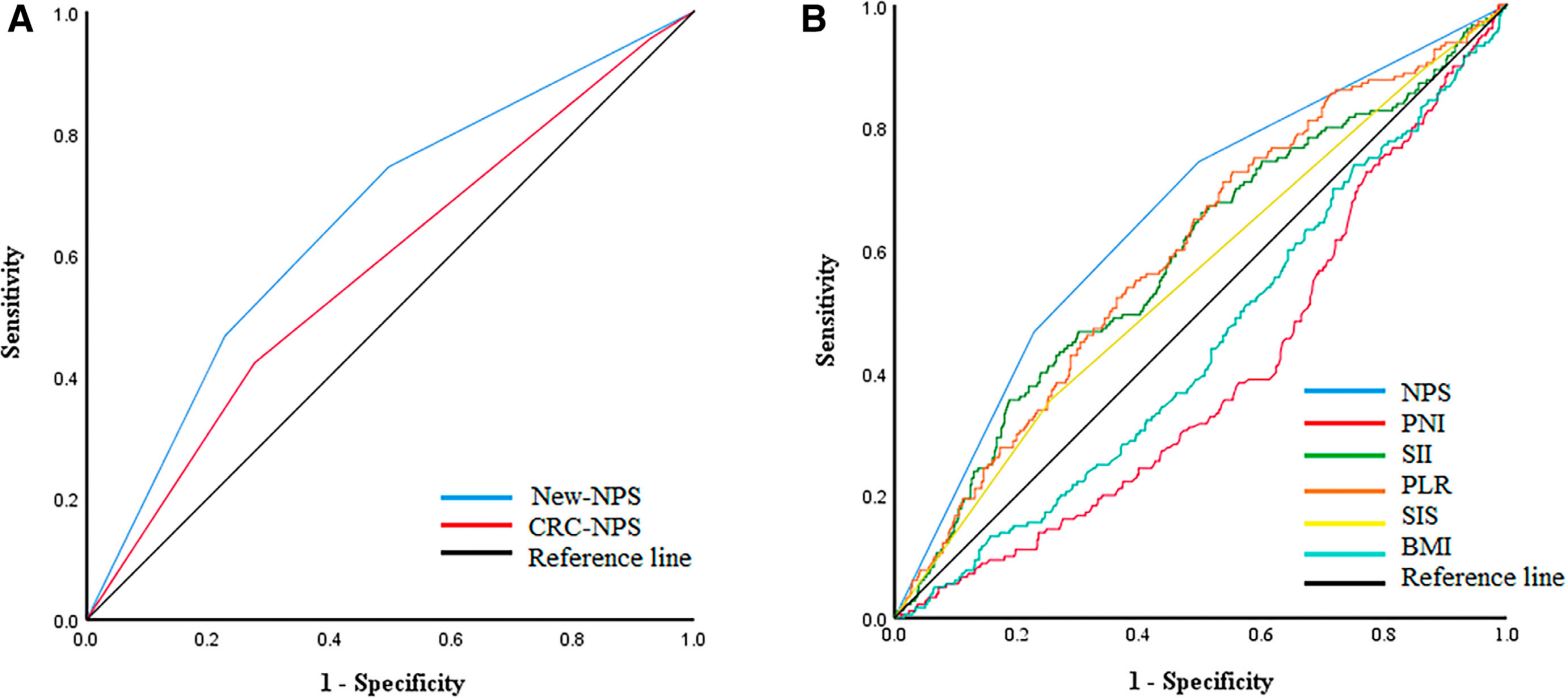
(A) Receiver operating characteristic (ROC) curves of the GC‐NPS and CRC‐NPS. (B) Receiver operating characteristic (ROC) curves of the NPS, SII, PLR, PNI, SIS, and BMI among all patients.

To verify whether New‐NPS is suitable for predicting the prognosis of GC patients, ROC showed that the AUC of New‐NPS was higher than CRC‐NPS (0.655 vs 0.578) (Figure 2A). This indicates that the New‐NPS reconstructed according to the immune and nutritional status of GC patients can better predict the prognosis of GC patients.

### 3.3 The value of the inflammation and nutritional indexes for the long‐term prognosis of patients

The AUC of NPS, SIS, PNI, SII, PLR, and BMI were 0.655 (95% CI: 0.609–0.701), 0.555 (95% CI: 0.506–0.604), 0.603 (95% CI: 0.351–0.443), 0.588 (95% CI: 0.539–0.637), 0.597 (95% CI: 0.550–0.645), and 0.555 (95% CI: 0.397–0.492) (Figure 2B). The AUC of NPS was higher than different prognostic markers. Thus, compared with different prognostic markers, NPS has higher accuracy in predicting OS.

### 3.4 ROC

**FIGURE 3:**
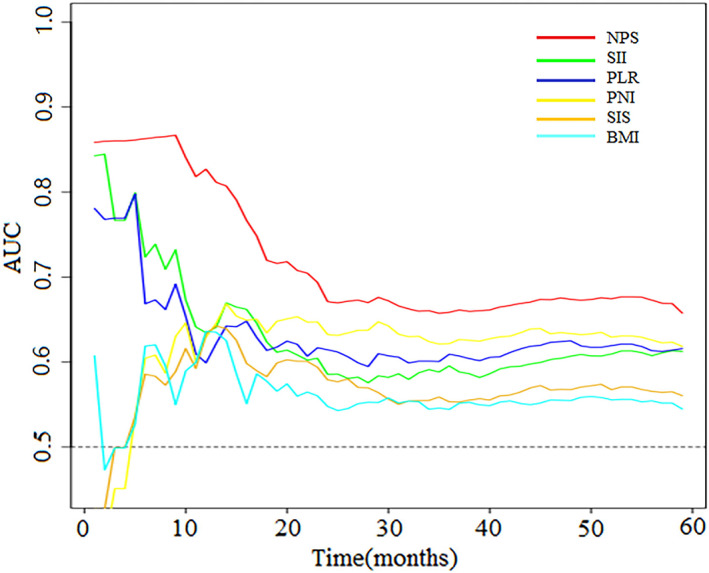
Time‐dependent ROC curves for the NPS, SII, PLR, PNI, SIS, and BMI. The horizontal axis represents the months after surgery, and the vertical axis represents the estimated AUC for survival at the time of interest.

We established a T‐ROC curve to compare the prognostic value of the NPS with different prognostic markers. During the entire observation period, the area under the curve of the NPS was higher than that of the SIS, PNI, SII, PLR, and BMI (1 year after surgery: 0.8270 vs. 0.6328, 0.6345, 0.5989, 0.6362), (3 years after surgery: 0.6584 vs. 0.5532, 0.6216, 0.5954, 0.6091, 0.5441) (Figure 3).

### 3.5 New‐NPS and patient survival

**FIGURE 4:**
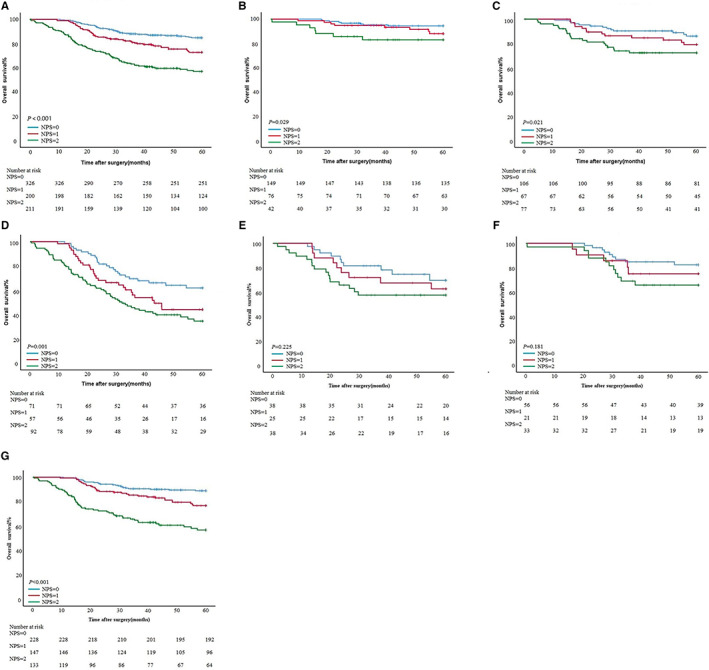
Survival curve subgroup analyses of patients. (A) Overall patients. (B) StageI. (C) StageII. (D) StageIII. (E) Upper third. (F) Middle third. (G) Lower third.

To compare the overall survival of the different NPS groups, the 5‐year OS rates of the patients was analyzed by Kaplan–Meier survival curve (Figure 4A). The 5‐year OS rates of patients with NPS = 0, NPS = 1, and NPS = 2 were 84.8%, 72.8%, and 57.0% (55.24 ± 0.69, 51.57 ± 1.11 and 43.36 ± 1.48, *P* < 0.001).

For different pTNM stages, the 5‐year survival rates of NPS = 0–2 in stage I patients were 94.4%, 88.0%, and 82.9% (58.13 ± 0.65，57.10 ± 1.14 and 52.45 ± 2.65, *P* = 0.029). The 5‐year survival rates of NPS = 0–2 in stage II patients were 86%, 79%, and 72.3% (56.06 ± 1.13, 53.82 ± 1.70 and 48.57 ± 2.23, *P* = 0.021). The 5‐year survival rates of NPS = 0–2 in stage III patients were 61.9%, 44.1%, and 34.6% (47.78 ± 2.06, 41.24 ± 2.53 and 35.22 ± 2.28, *P* = 0.001) (Figure 4B–D).

According to different tumor locations, the 5‐year survival rates of NPS = 0–2 in the upper third of the stomach patients were 69.7%, 62.7%, and 57.6% (50.78 ± 2.65, 47.34 ± 3.68 and 41.41 ± 3.63, *P* = 0.225). The 5‐year survival rates of NPS = 0–2 in the middle third of the stomach patients were 82.4%, 75.0%, and 65.8% (54.93 ± 1.60, 51.69 ± 3.32 and 48.41 ± 2.99, *P* = 0.181). The 5‐year survival rates of NPS = 0–2 in the lower third of the stomach patients were 88.9%, 76.7%, and 56.7% (56.30 ± 0.75, 53.21 ± 1.18 and 43.29 ± 1.91, *P* < 0.001) (Figure 4E–G).

Chi‐square analysis indicated that NPS had a statistically significant association with sex, age, BMI, CEA, CA19‐9, Borrmann type, tumor diameter, tumor location, pTNM stage, venous invasion, nerve infiltration, lymph node metastasis rate, vascular invasion, PNI, and SIS (*P* = 0.008, *P* = 0.002, *P* = 0.003, *P* = 0.009, *P* = 0.001, *P* < 0.001, *P* < 0.001, *P* = 0.041, *P* < 0.001, *P* = 0.001, *P* < 0.001, *P* < 0.001, *P* < 0.001, *P* < 0.001, and *P* < 0.001) (Table 3).

**TABLE 3:** Chi‐square analysis of New‐NPS and patient characteristics

Variable	NPS 0,1 (*n* = 326)	NPS 2 (*n* = 200)	NPS 3,4 (*n* = 211)	*P* value
Sex				**0.008**
Male	214 (65.6)	140 (70.0)	165 (78.2)	
Female	112 (34.4)	60 (30.0)	46 (21.8)	
Age (years)				**0.002**
≤60	197 (60.4)	115 (57.5)	95 (45.0)	
>60	129 (39.6)	85 (42.5)	116 (55.0)	
BMI (kg/m^2^)				**0.003**
≤22.8	141 (43.3)	105 (52.5)	122 (57.8)	
>22.8	185 (56.7)	95 (47.5)	89 (42.2)	
CEA ng/ml				**0.009**
≤5	291 (89.3)	175 (87.5)	169 (80.1)	
>5	35 (10.7)	25 (12.5)	42 (19.9)	
CA19‐9 U/ml				**0.001**
≤37	305 (93.6)	184 (92.0)	178 (84.4)	
>37	21 (6.4)	16 (8.0)	33 (15.6)	
Borrmann type				**<0.001**
0–II	198 (60.7)	96 (48.0)	100 (47.4)	
III	96 (29.4)	69 (34.5)	95 (45.0)	
IV	32 (9.8)	35 (17.5)	16 (7.6)	
Tumor diameter (mm)				**<0.001**
≤50 mm	254 (77.9)	142 (71.0)	103 (48.8)	
>50 mm	72 (22.1)	58 (29.0)	108 (51.2)	
Tumor location				**0.041**
Upper third	38 (11.7)	25 (12.5)	38 (18.0)	
Middle third	56 (17.2)	21 (10.5)	33 (15.6)	
Lower third	228 (69.9)	147 (73.5)	133 (63.0)	
Total stomach	4 (1.2)	7 (3.5)	7 (3.3)	
Pathological stage				**<0.001**
I	149 (45.7)	76 (38.0)	42 (19.9)	
II	106 (32.5)	67 (33.5)	77 (36.5)	
III	71 (21.8)	57 (28.5)	92 (43.6)	
Venous invasion				**0.001**
No	249 (76.4)	150 (75.0)	132 (62.6)	
Yes	77 (23.6)	50 (25.0)	79 (37.4)	
Neural infiltration				**<0.001**
No	188 (57.7)	90 (45.0)	88 (41.7)	
Yes	138 (42.3)	110 (55.0)	123 (58.3)	
Lymph node ratio (%)				**<0.001**
0	191 (58.6)	100 (50.0)	74 (35.1)	
0–0.3	104 (31.9)	65 (32.5)	87 (41.2)	
0.3–0.6	20 (6.1)	23 (11.5)	31 (14.7)	
>0.6	11 (3.4)	12 (6.0)	19 (9.0)	
Vascular invasion				**<0.001**
No	227 (69.6)	140 (70.0)	115 (54.5)	
Yes	99 (30.4)	60 (30.0)	96 (45.5)	
PNI				**<0.001**
≤50.83	48 (14.7)	97 (48.5)	173 (82.0)	
>50.83	278 (85.3)	103 (51.5)	38 (18.0)	
SIS				**<0.001**
0	86 (26.4)	13 (6.5)	1 (0.5)	
1	208 (63.8)	133 (66.5)	90 (42.7)	
2	32 (9.8)	54 (27.0)	120 (56.9)	

*Note*: Borrmann type and pTNM stage were determined according to the eighth edition of the AJCC Cancer Staging Manual. Tumor location and tumor diameter were determined according to the postoperative pathology report. Statistically significant *P* values are in bold (*P* < 0.05).

Abbreviations: BMI, body mass index; CEA, carcinoembryonciantigen; CA19‐9, carbohydrateantigen19‐9.

### 3.6 Analysis of univariate and multivariate factors affecting the prognosis of patients

The Cox proportional hazards regression model was performed to determine the independent risk factors. Univariate analysis showed that age (*P* = 0.024), BMI (*P* = 0.015), CEA (*P* < 0.001), CA19‐9 (*P* < 0.001), Borrmann type (*P* < 0.001), tumor diameter (*P* < 0.001), tumor location (*P* < 0.001), pTNM stage (*P* < 0.001), NPS (*P* < 0.001), PNI (*P* < 0.001), SIS (*P* = 0.014), venous invasion (P < 0.001), nerve infiltration (P < 0.001), lymph node metastasis rate (P < 0.001), vascular infiltration (P < 0.001), and postoperative chemotherapy (*P* = 0.002) were statistically significant. Multivariate analysis showed that CEA (*P* = 0.026), Borrmann type (*P* = 0.001), pTNM staging (*P* < 0.001), NPS (*P* < 0.001), and nerve infiltration (*P* = 0.035) were independent risk factors related to patient prognosis (Table 4).

**TABLE 4:** Prognostic factors of patients with GC by univariate and multivariate analyses based on Cox regression analysis

Characteristics				
	Univariate analysis		Multivariate analysis	
	HR (95% CI)	*P* value	HR (95% CI)	*P* value
Sex		0.904		
Male	1			
Female	0.981 (0.713–1.349)			
Age (years)	1.018 (1.002–1.034)	**0.024**	1.012 (0.995–1.028)	0.161
BMI (kg/m^2^)	0.945 (0.903–0.989)	**0.015**	0.979 (0.936–1.024)	0.361
CEA ng/ml	1.006 (1.003–1.009)	**<0.001**	1.004 (1.000–1.007)	**0.026**
CA19‐9 U/ml	1.002 (1.001–1.003)	**<0.001**	1.001 (1.000–1.001)	0.283
Borrmann type		**<0.001**		**0.001**
0–II	1		1	
III	2.611 (1.868–3.650)	**<0.001**	1.351 (0.933–1.957)	0.111
IV	3.796 (2.508–5.745)	**<0.001**	2.562 (1.570–4.182)	**<0.001**
Tumor diameter (mm)		**<0.001**		0.662
≤50 mm	1		1	
>50 mm	2.886 (2.152–3.870)		1.084 (0.756–1.554)	
Tumor location		**<0.001**		0.074
Upper third	1		1	
Middle third	0.573 (0.343–0.957)	0.033	0.508 (0.291–0.886)	**0.017**
Lower third	0.548 (0.374–0.803)	**0.002**	0.784 (0.522–1.176)	0.239
Total stomach	2.558 (1.352–4.838)	**0.004**	1.080 (0.532–2.193)	0.831
Pathological stage		**<0.001**		**<0.001**
I	1		1	
II	2.346 (1.422–3.871)	**0.001**	1.521 (0.815–2.839)	0.187
III	7.952 (5.070–12.472)	**<0.001**	4.528 (2.017–10.164)	**<0.001**
Naples		**<0.001**		**<0.001**
0 or 1	1		1	
2	1.883 (1.262–2.811)	**0.002**	1.790 (1.124–2.851)	**0.014**
3 or 4	3.706 (2.585–5.313)	**<0.001**	3.868 (2.364–6.331)	**<0.001**
PNI	0.953 (0.931–0.974)	**<0.001**	1.016 (0.981–1.052)	0.366
SIS		**0.014**		0.265
0	1		1	
1	1.191 (0.735–1.928)	0.478	0.867 (0.500–1.503)	0.611
2	1.794 (1.086–2.965)	**0.023**	0.625 (0.317–1.234)	0.176
Venous invasion		**<0.001**		0.891
No	1		1	
Yes	2.501 (1.864–3.355)		1.039 (0.604–1.787)	
Neural infiltration		**<0.001**		**0.035**
No	1		1	
Yes	3.882 (2.747–5.487)		1.597 (1.033–2.467)	
Lymph node ratio(%)		**<0.001**		0.368
0	1		1	
0–0.3	2.194 (1.516–3.175)	**<0.001**	0.682 (0.392–1.187)	0.176
0.3–0.6	5.107 (3.276–7.682)	**<0.001**	0.744 (0.372–1.486)	0.402
>0.6	6.980 (4.325–11.266)	**<0.001**	0.944 (0.453–1.969)	0.878
Vascular invasion		**<0.001**		0.379
No	1		1	
Yes	2.727 (2.032–3.660)		1.291 (0.731–2.283)	
Postoperative chemotherapy		**0.002**		0.396
No	1		1	
Yes	1.605 (1.197–2.152)		0.865 (0.619–1.209)	

*Note*: Borrmann type and pTNM stage were determined according to the eighth edition of the AJCC Cancer Staging Manual. Tumor location and tumor diameter were determined according to the postoperative pathology report. Statistically significant *P* values are in bold (*P* < 0.05).

Abbreviations: BMI, body mass index; CEA, carcinoembryonciantigen; CA19‐9, carbohydrateantigen19‐9.

### 3.7 A nomogram for assessing patient prognosis

**FIGURE 5:**
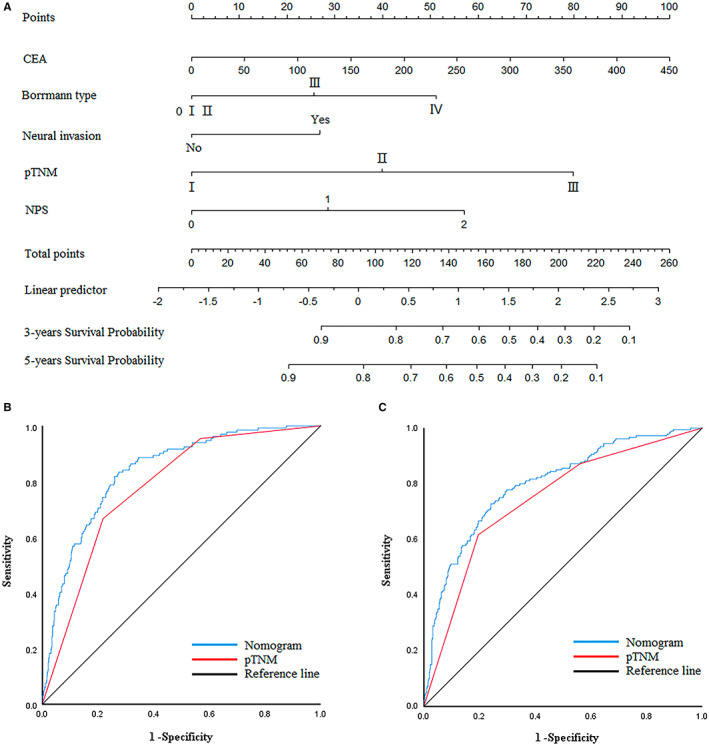
(A) Nomogram model predicting the 3‐ and 5‐year survival of all patients. (B) ROC curve of the nomogram model predicting the 3‐year survival of all patients. (C) ROC curve of the nomogram model predicting the 5‐year survival of all patients.

**FIGURE 6:**
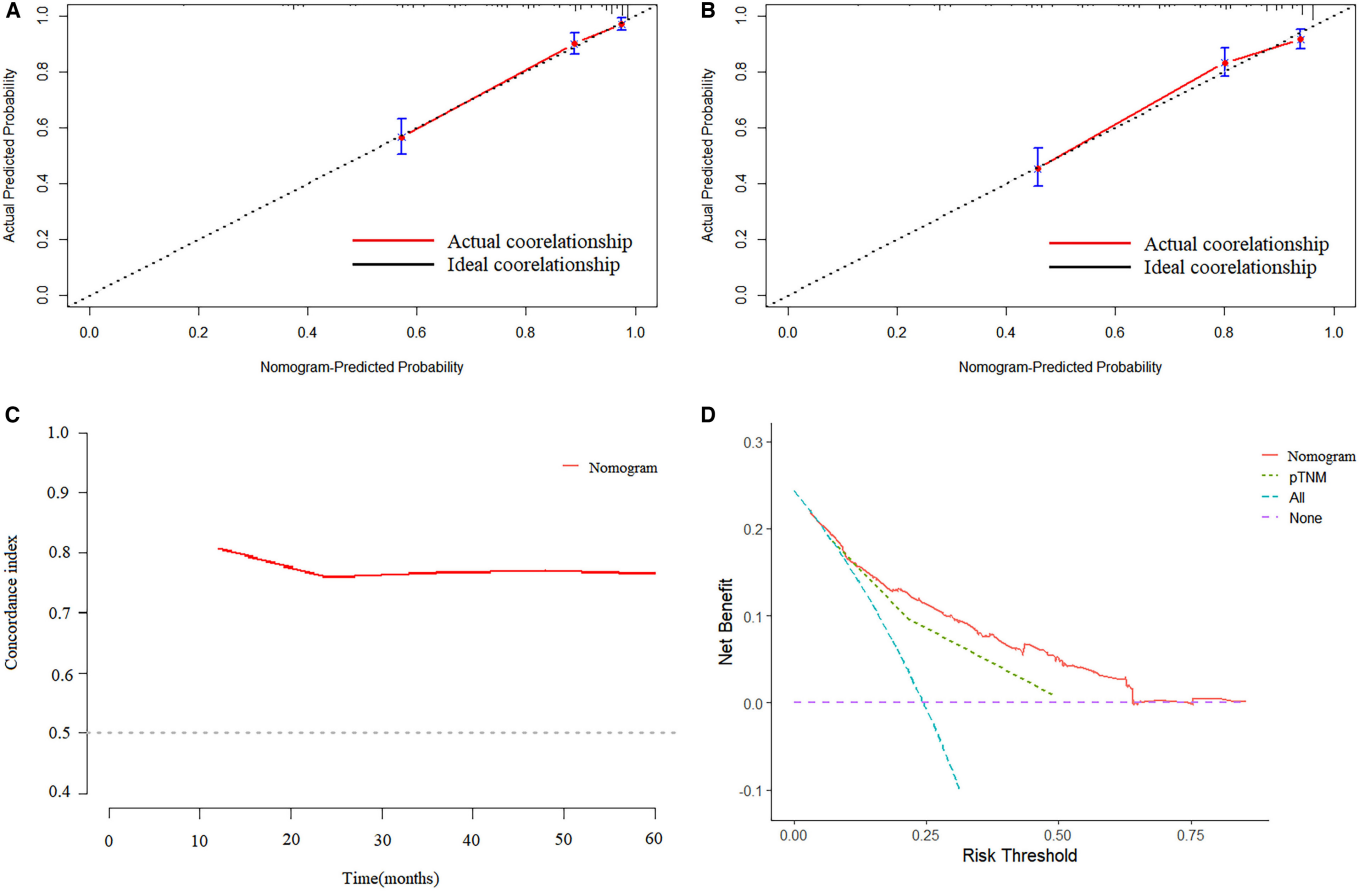
(A,B) Calibration plots for the nomogram. Correlationship between the predicted probabilities based on the nomogram and actual values. (A) 3 years. (B) 5 years. (C) Concordance Index of Nomogram. (D) Decision curve analysis for 5‐year survival prediction.

Since CEA, Borrmann type, pTNM staging, and nerve infiltration were independent risk factors related to patient prognosis, and then, we combined these factors to construct a nomogram for predicting the prognosis patients (Figure 5A). The AUC for predicting the three‐year and five‐year prognosis of GC patients was 0.836 (95% CI: 0.801–0.871) and 0.799 (95% CI: 0.761–0.837), respectively. The sensitivity was 0.833 and 0.728, and the specificity was 0.722 and 0.756, respectively (Figure 5B,C). Calibration plots showed that nomograms performed well in predicting patients' OS at 3 and 5 years (Figure 6A,B). The c‐index of the nomogram was 0.766 (Figure 6C). Decision curves showed that the nomogram has a higher overall net benefit than pTNM over the threshold range (Figure 6D).

### 3.8 Verify the suitability of New‐NPS

**FIGURE 7:**
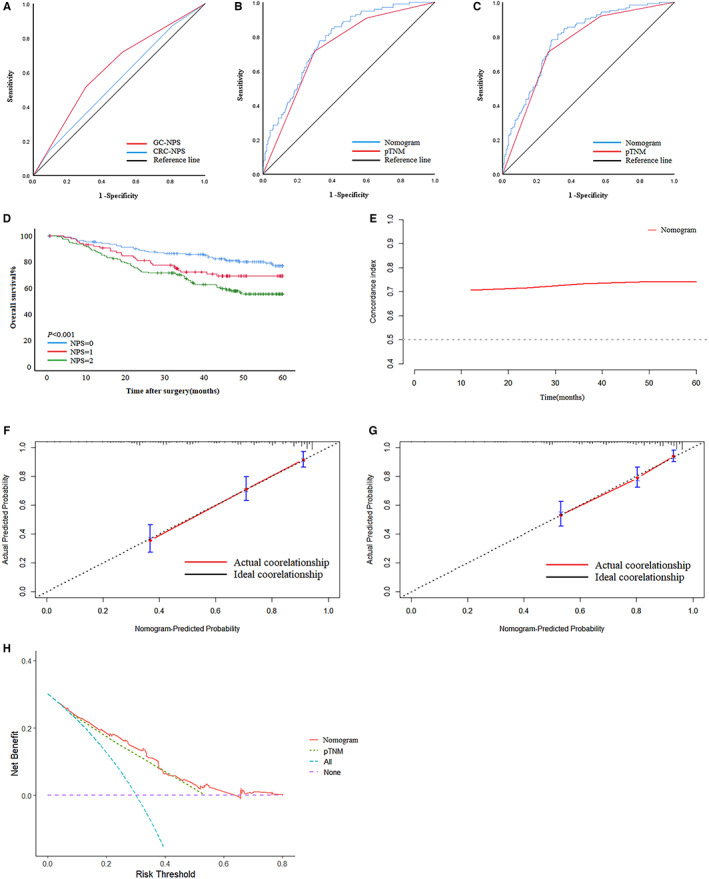
Results of New‐NPS in the validation cohort. (A) Receiver operating characteristic (ROC) curves of the GC‐NPS and CRC‐NPS. (B) ROC curve of the nomogram model predicting the 3‐year survival of all patients. (C) ROC curve of the nomogram model predicting the 5‐year survival of all patients. (D) Survival curve subgroup analyses of overall patients. (E) Concordance Index of Nomogram. (F,G) Calibration plots for the nomogram. (F) 3 years. (G) 5 years. (H) Decision curve analysis for 5‐year survival prediction.

The validation cohort showed that the AUC of New‐NPS was higher than CRC‐NPS 0.623 (95%CI: 0.564–0.682) vs 0.549 (95%CI: 0.489–0.609) (Figure 7A). The 5‐year survival rates of NPS = 0–2 in the validation cohort were 76.5%, 65.6%, 54.5% (*P* < 0.001) (Figure 7D). The AUC of the nomogram for predicting the three‐year and five‐year prognosis of GC patients was 0.775 and 0.788, the sensitivity was 0.847 and 0.782，the specificity was 0.601 and 0.714 (Figure 7B,C). The c‐index of the nomogram was 0.742 (Figure 7E). Calibration plots showed that nomograms performed well in predicting patients' OS at 3 and 5 years (Figure 7F,G). Decision curves showed that the nomogram has a higher overall net benefit than pTNM over the threshold range (Figure 7H). These results show that the New‐NPS constructed according to the nutritional and immune status of GC patients in our institution has certain applicable value and is worthy of clinical application.

## 4 DISCUSSION

NPS was proposed by Galizia et al. in CRC which is composed of preoperative LMR and NLR, Alb, TC and contains the inflammatory and nutritional status.^7^ However, our study found that there were some differences in the immune and nutritional status between GC and CRC, which led to the optimum cutoff values for NPS indicators in GC being different from CRC. Therefore, we reconstructed the New‐NPS according to the nutritional status in GC patients.

As two different solid tumors of the digestive tract, there are some differences in tumor microenvironment immune between GC and CRC, this may be related to the different functions of infiltrating immune cells in the TME. In GC, TANs secrete IL‐17a through the ERK pathway to promote the progression and metastasis of tumor cells.^21^ However, in CRC, TANs activate TGF‐β by secreting MMP9 and inhibit T cell function, leading to CRC progression and metastasis.^22^ Furthermore, due to the high heterogeneity of GC, the detection of immune infiltration in pathological sections by immunohistochemistry is limited. The tumor cells in the TME may cause immune activation after entering the peripheral blood, leading to an increase in the inflammatory response which can lead to different degrees of inflammation in the body. Compared with immunohistochemical, the inflammation index is convenient for operation and has been verified in clinical practice. Neal et al. found that the optimal cutoff value of LMR was 2.35 in CRC while Zhou et al. found that the cutoff value was 4.32 in GC.^23^, ^24^ This further indicates that there is a difference between CRC and GC in peripheral blood immunity. In addition, GC occurs in the upper digestive tract may cause a series of clinical symptoms, such as vomiting, regurgitation, dysphagia, and malignant obstruction, that lead to nutrient intake and absorption disorders.^12^ By contrast, CRC occurs in the lower digestive tract has a limited impact on nutrient intake and absorption.^13^ This may explain McKenna et al.’s suggestion that malnutrition varies widely by cancer type and that weight loss is more common in gastric cancer than in other cancers.^6^ In clinical practice, radical gastrectomy is the main treatment for gastrointestinal tumors. However, GC and CRC patients have different gastrointestinal reconstruction methods and scopes of lymph node dissection, which makes GC patients prone to weight loss after gastrectomy (approximately 5%–15%) and malnutrition.^25^

Although we have found some differences in the immune and nutritional status of CRC and GC patients. However, previous studies still show that the CRC‐NPS can effectively predict the prognosis of GC,^10^, ^26^, ^27^, ^28^, ^29^ this indicates that CRC‐NPS has good applicability. However, there may be some differences in the nutritional status of patients in different regions and different populations. The prevalence of overweight and hypertriglyceridemia was higher in the north compared with south China, and in urban compared with rural regions,^30^ and our institution is located in Northeast China, which also has a higher prevalence of obesity than South and Southwest.^7^ Worldwide, the prevalence of obesity was higher in Western compared with Asia.^8^, ^31^ Furthermore, the prevalence of obesity also varies among different racial populations.^32^ The same nutritional assessment criteria may not be able to accurately assess the nutritional status of patients in different regions.^33^ Therefore, specific nutritional assessment tools have also been generated for different populations, such as, Geriatric Nutrition Risk Index (GNRI) for elderly patients,^34^ Obesity Risk Index (ORI) for obese genetic patients.^35^ As with most clinical retrospective analyses, there may be some limitations for the generalizability for clinical guidance of our results, but this does not prevent us from making a suggestion for more gastroenterologists: different countries, different dietary habits, and different physiques of GC patients need different evaluation criteria for nutritional status evaluation. We also call for more multi‐center, big‐data studies to use the different regions of patients as an independent evaluation factor. Therefore, our study adopted the optimal cutoff values for LMR, NLR, Alb, and TC to specialized construct the New‐NPS to better serve GC patients in this region. Although our New‐NPS may not be applicable to GC patients in different institutions, the trend of this study suggests that specific prognostic scores need to be constructed according to the nutritional status of GC patients in different regions to accurately assess the prognosis of patients.

Inflammatory cells can promote tumor progression through different mechanisms, such as immunosuppression and matrix remodeling.^36^ The inflammatory indexes calculated from peripheral blood immune cells can accurately predict the prognosis of patients and has been widely used in clinical practice.^24^, ^37^ The cutoff values of the NLR and LMR used by Galizia et al. for CRC were 2.96 and 4.44, while the cutoff values of the NLR and LMR were 2.37 and 3.10 in our study. These differences are probably due to immune differences generated by the different tumor lesions.^38^ Furthermore, the cutoff values of NLR and LMR are different from Wang er al.’s NLR in stage IV GC (2.37 vs. 2.8) and Zhou et al.’s LMR in the stage II/III GC (3.10 vs 4.32).^24^, ^37^ This difference may be due to more stage I patients in our study. It also indicates that the inflammatory response in the body of these patients may be aggravated by the progression of pTNM by stages. Lymphocytes can induce cytotoxic cell death and produce inhibitory cytokines to inhibit tumor cell proliferation, and a decrease in lymphocytes can also cause immunosuppression and lead to cancer progression.^36^, ^37^, ^38^ Neutrophils promote angiogenesis and inhibit the immune function of T lymphocytes.^39^ Tumor‐associated macrophages (TAMs) promote tumor progression by producing cytokines such as IL‐10.^40^ The combined inflammatory index can provide more comprehensive prognostic information for patients, Wang et al. combined NLR and PLR and achieved good application,^37^ which also provided a good theoretical basis for the integration of NLR and LMR to construct the NPS. In addition, the dynamic changes in the inflammation index will also help to predict the prognosis of patients. Yin et al. found that the dynamic changes in the PLR could evaluate the sensitivity of GC patients to XELOX chemotherapy.^41^ Therefore, our follow‐up studies may predict the prognosis of patients based on the dynamic changes in the NPS before and after surgery.

Malnutrition is related to the highly aggressiveness of tumors.^42^ Due to the high incidence of clinical symptoms such as reflux and malignant obstruction, and radical gastrectomy removes at least two‐thirds of the stomach and omentum and D2 or D2+ lymph node dissection, these factors may directly increase the nutritional burden of GC patients, these mechanisms may explain that the cutoff value of TC for GC is lower than that for CRC (156.81 vs. 180). Postoperative weight loss in GC patients is associated with fat loss,^43^ this also suggests that lower total TC levels may be a more common cause of weight loss in GC patients than in CRC patients. TC participates in and controls the fluidity of the cell membrane and affects signal transmembrane conduction,^44^ and the decreased levels of TC suggest a poor prognosis.^45^ Alb has the properties of stabilizing cell growth and resisting tumor oxidation, and the reduction in Alb levels suggests decreased body defenses, thus leading to poor prognosis.^46^, ^47^, ^48^ In addition, we found that NPS had the highest AUC, which also indicated that NPS was more accurate in predicting the prognosis of patients and could provide more clinical information. Therefore, the nutritional and inflammatory status of the patient should be comprehensively considered in clinical work to more accurately predict the prognosis of patients.

In this study, we found that the New‐NPS was an independent risk factor for patient prognosis. This finding showed that NLR, LMR, Alb, and TC play an important role in GC patients. Chi‐square analysis indicated that as the total score of NPS increased, the levels of CEA and CA19‐9 in the patient's body gradually increased and the proportion of nerve infiltration, venous invasion, vascular invasion, and lymph node metastasis rate gradually increased, these factors have also been confirmed to be risk factors that are related to prognosis.^49^, ^50^, ^51^ This finding also suggests that increased inflammation or a decreased nutritional status will cause the tumor to be more aggressive and lead to a poor prognosis. For different tumor locations, patients with tumors located in the upper third of the stomach had a worse prognosis than those in the lower third of the stomach. The reason for this phenomenon may be that the upper third of the stomach contains the cardia and fundus of the stomach. Kim et al. showed that cardia cancer is more aggressive than non‐cardia cancer.^52^ This also suggests that patients with cardia cancer may be accompanied by significantly elevated inflammation and low nutritional status. Therefore, additional attention should be given to such patients in the clinical setting, and reasonable supportive treatment before surgery may improve the prognosis of patients.

Clinically, pTNM stage provides useful but incomplete prognostic information for prognosis. GC is a highly heterogeneous malignant tumor of the digestive tract, and this heterogeneity also leads to differences in the prognosis of patients.^53^, ^54^ Therefore, in order to accurately judge the prognosis, combining the macroscopic basis of pTNM staging with microscopic hematological indicators can provide clinicians with more useful information. Liu et al. constructed a nomogram using the comprehensive score (SPS) of inflammatory parameters and nutritional parameters, tumor location and pTNM stage that can better predict the prognosis of stage II‐III gastric cancer patients undergoing postoperative chemotherapy.^55^ Fang et al. constructed a nomogram based on SII to predict prognosis in stage I‐II GC.^56^ In our study, according to multivariate analysis, CEA, Borrmann type, New‐NPS, pTNM stage, and nerve infiltration were independent factors related to the prognosis of GC patients. Then, combined with the above indicators, a nomogram model was constructed to predict the prognosis of the corresponding patients. ROC analysis found that the AUC of this nomogram for predicting the 3‐year and 5‐year prognosis of GC patients was higher than the AUC of pTNM (0.837 vs. 0.778, 0.799 vs. 0.743), and the nomogram model is well validated in the validation cohort. Therefore, the predictive model constructed by macroscopic TNM stage and the microscopic NPS can effectively predict the prognosis of GC patients, and it is worthy of further clinical verification and promotion.

## 5 RESEARCH LIMITATIONS

As a retrospective study, there are still some limitations to this study. First, this study is a single‐center study of Asian patients, and whether the results are suitable for all GC patients still requires a larger sample size. Second, the cutoff values of the inflammatory parameters and nutritional parameters in the peripheral blood before surgery may change with the number of patients. Finally, the cutoff values of inflammatory parameters and nutritional parameters in different studies may be different; therefore, it may not be possible to unify the scoring standard for the NPS for GC patients. However, the trend of this study suggests that reconstructing the NPS based on the inflammation and nutritional status of GC patients can better serve GC patients.

### 5.1 Conclusion

The New‐NPS constructed based on the cutoff values of preoperative NLR, LMR, Alb, and TC that was calculated based on the immune and nutritional status of GC patients can better predict the prognosis of GC patients. The New‐NPS has a higher accuracy than different prognosis markers, and NPS = 2 indicates a poor prognosis. New‐NPS is an independent factor of the prognosis of patients with GC, which can be combined with clinicopathological features to construct a nomogram to predict the prognosis.

## AUTHOR CONTRIBUTIONS

Hao Wang and Tianyi Fang designed and conceived this project, they contributed equally to this work. Hao Wang, Tianyi Fang, Xin Yin, Shenghan Lou, Bangling Han, and Jialiang Gao interpretated and analyzed the data. Yingwei Xue revised the manuscript for important intellectual content. Hao Wang, Tianyi Fang, Xin Yin, Yufei Wang, Daoxu Zhang, Xibo Wang, Yimin Wang, and Yao Zhang participated in the patient information collection. All authors read and approved the final manuscript.

## FUNDING INFORMATION

This work was supported by the Nn10 program of Harbin Medical University Cancer Hospital, China (No. Nn10 PY 2017‐03) and the Harbin Science and Technology Bureau Research and Development Project of Applied Technology (No. 2017RAXXJ054).

## CONFLICT OF INTEREST

The authors declare that they have no conflict of interest.

## ETHICS STATEMENT

All programs followed were according to the ethical standards of the Human Subjects Responsibility Committee (institutions and countries), as well as the 1964 Helsinki Declaration and subsequent editions. This research was approved by the Ethics Committee of the Harbin Medical University Cancer Hospital (Approval Number: 2019‐57‐IIT).

## Data Availability

Patients’ data were saved in the Gastric Cancer Information Management System v1.2 of Harbin Medical University Cancer Hospital (Copyright No.2013SR087424, http: www.sgihmu.com).
